# Multiscale Stem Cell Technologies for Osteonecrosis of the Femoral Head

**DOI:** 10.1155/2019/8914569

**Published:** 2019-01-08

**Authors:** Yi Wang, Xibo Ma, Wei Chai, Jie Tian

**Affiliations:** ^1^Department of Orthopedics, Chinese PLA General Hospital, Beijing 100853, China; ^2^CAS Key Laboratory of Molecular Imaging, Institute of Automation, Chinese Academy of Sciences, Beijing 100190, China; ^3^The University of Chinese Academy of Sciences, Beijing 100049, China

## Abstract

The last couple of decades have seen brilliant progress in stem cell therapies, including native, genetically modified, and engineered stem cells, for osteonecrosis of the femoral head (ONFH). In vitro studies evaluate the effect of endogenous or exogenous factor or gene regulation on osteogenic phenotype maintenance and/or differentiation towards osteogenic lineage. The preclinical and clinical outcomes accelerate the clinical translation. Bone marrow mesenchymal stem cells and adipose-derived stem cells have demonstrated better effects in the treatment of femoral head necrosis. Various materials have been used widely in the ONFH treatment in both preclinical and clinical trials. In a word, in vivo and multiscale efforts are expected to overcome obstacles in the approaches for treating ONFH and provide clinical relevance and commercial strategies in the future. Therefore, we will discuss the above aspects in this paper and present our opinions.

## 1. Introduction

Osteonecrosis of the femoral head (ONFH) is a debilitating skeletal disorder leading to loss of hip joint function which brings a heavy financial burden to healthcare system worldwide [1, 2]. The repair processes following osteonecrosis include the differentiation of preexisting mesenchymal stem cells (MSC) (the latest research shows that osteocytes are differentiated from skeletal stem cells (SSC) [3]) into osteoblasts, bone matrix secretion, and mineralization. The rate of bone generation is less than that of bone resorption, which will lead to a natural repair failure in the necrotic zone of the femoral head [4]. As a strategy to manage ONFH in the early stage, conservative treatments (e.g., physical therapy or pharmacotherapy) have questionable efficiency in current clinical practice [5–9]. For patients in the end stage of ONFH, total hip arthroplasty (THA) remains an inevitable choice as the clinical gold standard. However, THA has its disadvantages including the limited longevity of implants [10] and complications of surgical intervention (e.g., infection, revision, and dislocation) [11–13]. These disadvantages have triggered a growing expectation for research on femoral head regeneration.

**Figure 1:**
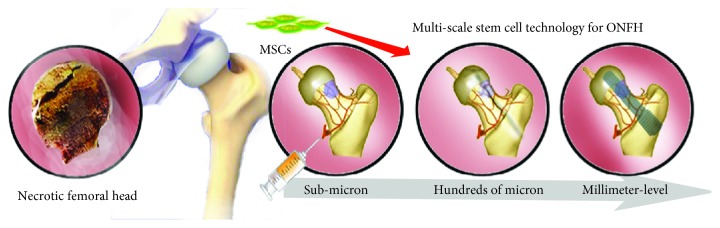
Multiscale stem cell technologies for ONFH therapies.

Stem cells have characteristics of proliferation and differentiation. These properties make stem cell technology stand out in the field of femoral head regeneration. In recent years, stem cell science has overcome many obstacles in ONFH treatments by using multiscale stem cell technologies [14]. Multiscale stem cell technology refers to the spatial scales of different stem cells alone or with material stem cells for treatment. In this review, we cover multiscale stem cell technologies to treat ONFH (Figure 1). We briefly review the changes affecting repair abilities of MSC in the osteonecrosis area and five main microRNAs about osteogenesis. We also discuss multiscale stem cell technologies to introduce new therapeutic strategies for ONFH therapies. The multiscale stem cell technologies cover micron-sized stem cell suspensions, tens to hundreds of micron-sized stem cell carriers, and millimeter-scale stem cell scaffolds. We also outline promising stem cell materials for bone regeneration in other fields and analyze their reference to this field. Finally, we discuss the future trends of multiscale stem cell technology for treatment of ONFH.

Mesenchymal stem cells can regenerate the necrotic area of the femoral head by multiscale stem cell technologies. The stem cells are delivered to the necrosis zone by injecting suspension into the lateral artery of the circumflex (submicron), by load on carriers via core decompression (hundreds of microns), and by load on scaffolds via implantation (millimeter-level).

## 2. Changes in Microenvironment and MicroRNAs

The pathophysiology of ONFH remains unclear, although many attempts have been made to establish theoretical models [15]. Several recognized risk factors of ONFH have been studied at the cellular or molecular biology level in recent years including traumatic factors (e.g., femoral neck/head fracture, dislocation of the hip, and femur skull slip) and nontraumatic factors (e.g., glucocorticoids, alcohol abuse, sickle cell disease, and lipid disorders) [16].

**Figure 2:**
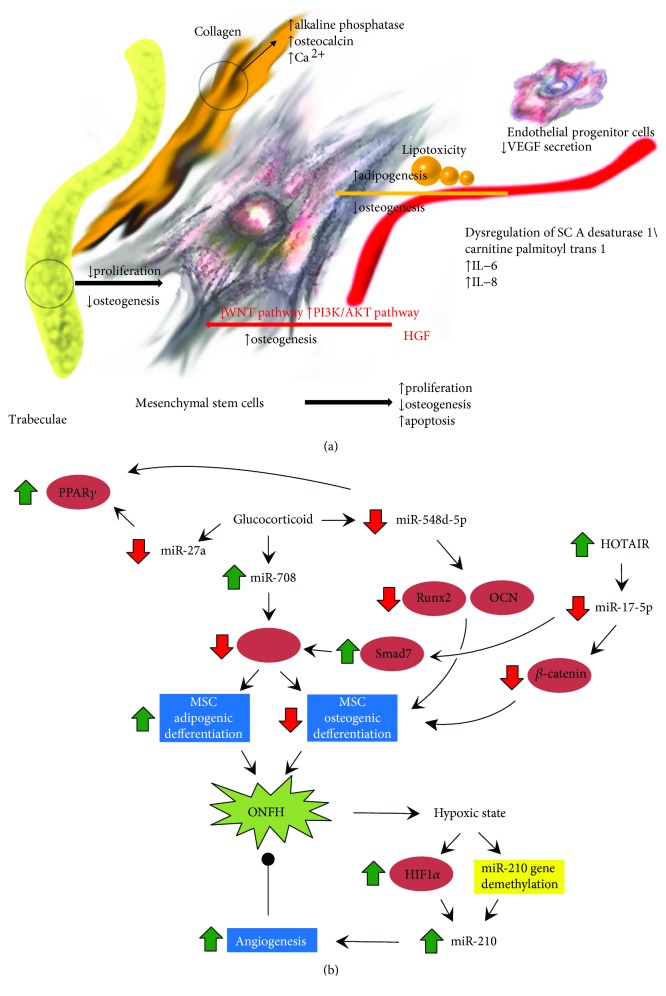
(a) Changes in proliferation and osteogenesis of stem cells in the area of osteonecrosis. (b) Five specific micro-RNAs play functional roles in femoral head necrosis. Figure adapted from ref. [103], John Wiley & Sons Publishers Ltd.

MSC extracted from necrotic trabeculae present decreased proliferation and osteogenesis [17]. However, the components around MSC have different effects on their activities (Figure 2(a)). The trabecular structure from the necrotic area promotes MSC proliferation but inhibits ossification [18], while the surrounding demineralized matrix can promote MSC ossification [19]. The colony-forming ability of endothelial progenitor cells in peripheral blood vessels decreases, and the ability to secrete the vascular endothelial growth factor (VEGF) also decreases which will result in no blood supply in the necrotic area and necrosis aggravation [20]. Lipotoxicity is a major factor of steroid-induced necrosis of the femoral head. Increased levels of palmitate and oleate lead to the dysregulation of stearoyl-coenzyme A desaturase 1/carnitine palmitoyl transferase 1 as well as increased expression of interleukin-6 and interleukin-8 (IL-6 and IL-8) which promote adipogenesis and inhibit osteogenesis [21]. The hepatocyte growth factor (HGF) promotes osteogenesis by activating the PI3K/AKT pathway and inhibiting the WNT pathway [22].

In addition, changes in microRNA (miR) expression of MSC in necrotic areas also play important roles in the progression of ONFH (Figure 2(b)). miR-708 is significantly upregulated in MSC from patients with steroid-induced ONFH. Targeting miR-708 enhances osteogenesis and inhibits adipogenesis of MSC, while knockdown of miR-708 avoids the inhibition of osteogenesis of glucocorticoids [23]. miR-210 demethylation promotes miR-210 expression and increases endothelial cell viability and differentiation for angiogenesis [24]. Overexpression of miR-548d-5p enhances the expression of osteocalcin and Runx2 (osteogenic transcription factor) and alkaline phosphatase (ALP). Thus, it can be a marker for osteoblast differentiation. Peroxisome proliferator-activated receptor gamma (PPAR*γ*) has been identified as a target for miR-548d-5p [25]. Downregulation of HOTAIR (HOX transcript antisense RNA), a long noncoding RNA, increases miR-17-5p levels and inhibits Smad7 expression. Knockout of HOTAIR promotes the expression of COL1A1, Runx2, and ALP [26, 27]. Both PPAR*γ* and gremlin 1 (GREM1) are the direct targets of miRNA-27a, and the knockdowns of them also enhance the osteogenesis of MSC [28]. MicroRNA activation or silence may be an effective method for the osteogenesis recovery of stem cells. However, the biosecurity still requires significant experimental evidence. Changes in or around stem cells can affect cytoactivity, and hence the therapeutic effects will be unstable. Therefore, the supplementary cells with healthy states are prerequisites for stem cell therapies.

## 3. Multiscale Stem Cell Technologies for ONFH Therapies

Over the past two decades, lots of efforts for femoral head regeneration have been made on cell-based repair in ONFH treatments. A variety of MSC have been used to regenerate the necrotic bone tissue with angiogenesis, including bone marrow mesenchymal stem cells (BMSC) [29], adipose-derived mesenchymal stem cells (ADSC) [30], synovial-derived mesenchymal stem cells (SDMSC) [31], dental-pulp stem cells (DPSC) [32], blood-derived mesenchymal stem cells (BDMSC) [33], and umbilical cord-derived mesenchymal stem cells (UCDMSC) [34]. However, the clinical translations of these methods are mainly limited by the following: (1) the method of cell implantations alone is only effective for patients in the early stage of ONFH [35]; (2) there is lack of safe, robust in vivo stem cell tracing techniques [36, 37]; (3) potential risks of heterotopic ossification [38] or tumorigenesis still exist [39]; and (4) the volume of tissue available to extract stem cells is limited. In addition, the stem cell implantation approach will influence the therapeutic and adverse effects of ONFH.

Stem cells alone cannot work well in the advanced stage of ONFH. Carriers or scaffolds loaded with stem cells can provide mechanical support while delivering stem cells to the necrosis zone. Better outcomes may be achieved by those methods.

Notably, the lack of control of cells following transplantation is another crucial issue in the biological-environmental field. One theory is that the implanted stem cells form new bones at the transplant site [40]. Another argument is that most of the stem cells do not differentiate into new osteoblasts, but play a regulation role by paracrine effects [41, 42]. Thus, the stem cell therapeutic process is expected to be monitored and the developments of cell tracing have skyrocketed in recent years. However, the large animal in vivo tracings are still limited by tracer properties (e.g., limited penetration depth of fluorescent dye, low sensitivity response of magnetic probe, or high biotoxicity). Safe, robust, and effective tracers are still underdeveloped [43, 44].

The risk of heterotopic ossification and tumor formation may be related to the choice of stem cell populations or subpopulations [45, 46]. Shimono et al. believed that mesenchymal stem cells with inhibited nuclear retinoic acid receptor-*γ* did not undergo heterotopic ossification [46]; the risk of tumor formation might be also reduced by selecting stem cell subpopulations without tumorigenicity [47, 48]. Therefore, the right choice of the subpopulation of stem cells may avoid these adverse effects.

The number of stem cells can influence the therapeutic effect [49]. The ability of stem cell proliferation can be enhanced by in vitro pretreatment [50], including transgene [51], drugs [52], and proliferation *in vitro* [53].

**Figure 3:**
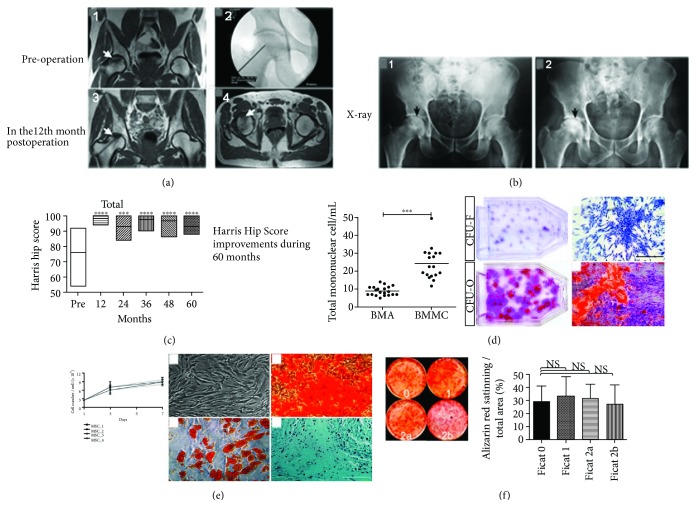
(a, 1) MRI T1-weighted (white arrow) suggests femoral head necrosis; (a, 2) X-ray guided minimally invasive decompression and implantation of MSC; (a, 3-4) MRI T1-weighted (12 months post-operation) shows femoral head edema decreased in the coronal plane (a, 3) and the axial plane (a, 4). (b, 1) Preoperative pelvic X-ray shows the necrosis zone; (b, 2) pelvic X-ray (60 months post-operation) shows the bone sclerosis band in the femoral head. (c) Harris Hip Score improvement with MSC therapy. (d) Concentrated MSC are of fibrogenic differentiation and osteogenic colony forming ability. (e) The proliferated MSC are of good differentiation potential of the three lineages. (f) The osteogenesis of MSC does not decline. Figure adapted from ref. [35].

Compared to intravascular infusion [54, 55], the method of in situ cell suspension implantation via core decompression (CD) can overcome obstacles of insufficient cell number due to cell redistribution. CD can also remove the necrotic tissue which is bad for osteogenesis. All in all, CD appears to be more reliable in increasing cell seeding density without the redistribution in blood. Therefore, in situ injection or transplantation of therapeutic cells can significantly improve the therapeutic efficacy (Figure 3).

In the remainder of this section, we will discuss how multiscale stem cell technology addresses the four major limitations and the delivery of stem cells.

### 3.1. Cell-Based Therapy Strategy

The selection of cell population initializes the stem cell therapy. Proper pretreatments can effectively improve survivability, proliferation ability, and/or osteogenic capability of MSC.

#### 3.1.1. Bone Marrow Stem Cells and Pretreatments

BMSC are the most common cell seeds in the treatment for ONFH. Many studies demonstrate the factors that influence osteogenic differentiation of BMSC to different degrees (e.g., growth factors, hormones, and small molecule drugs). HGF influences BMSC in a dose-dependent manner. The osteogenic differentiation of BMSC is promoted at a low concentration (20 ng/mL), by upregulated c-Met, p27, Runx2, and osterix, while BMSC are proliferated at a high concentration of HGF (100 ng/mL) by the overactivated ERK1/2 signaling pathway [29]. The granulocyte colony-stimulating factor and stem cell factor (G-CSF/SCF) promote the osteogenesis of BMSC and inhibit the caspase-3-dependent apoptosis [56]. P-glycoprotein (P-gp) alone or induced by icariin can alleviate oxidative stress of BMSC and promote osteogenesis [50, 57]. Lithium chloride (LiCl) promotes the osteogenesis of BMSC by inhibition on adipogenesis. The expression of PPAR*γ* and fatty acid-binding protein 4 (Fabp4) presents antiadipogenic effects associated with upregulated *β*-catenin and downregulated phosphorylated GSK-3*β* at the Tyr-216 site [58]. The decreased VEGF-D expression may have disastrous consequences for vascularization, while VEGF-A or B expresses at a normal level in ONFH patients [59]. Vitamin E shows protective effects against apoptosis by downregulating caspase-3 expression and upregulating Bcl-2 expression [52]. Vitamin K2 antagonizes steroids and promotes bone formation and bone morphogenetic protein (BMP) expression [60].

The osteogenic potential of BMSC can be enhanced when transfected into the *Homo sapiens* forkhead box C2 (Foxc2) gene via lentivirus [61]. In view of the effects of HGF on proliferation and differentiation of BMSC [29], BMSC transfected by the HGF gene appear to be a promising candidate for ONFH treatment [62]. BMSC can be endowed with the secretion of VEGF following the delivery of the VEGF 165 gene into cells by Lipofectamine. Thus, BMSC have the dual ability to promote osteogenesis and angiogenesis [51]. Based on this idea, BMSC infected by adeno-associated virus loaded with the VEGF 165 gene, BMP-7 gene, and internal ribosome entry site present enhanced osteogenesis and angiogenesis [63–66]. The specific siRNA can silence PPAR*γ*. The siRNA adenovirus vector can efficiently inhibit BMSC adipogenic differentiation induced by steroid, and BMSC retain their osteogenic potential by targeting PPAR*γ* [67].

#### 3.1.2. Preclinical and Clinical Trials

Intra-arterial or intravenous infusion of stem cells was declared as a safe and curative method for the ONFH [68]. Tong and his fellows performed the femoral circumflex arterial perfusion of autologous BMSC in canine models of ONFH established by the liquid nitrogen freezing method. The results showed that this strategy promoted vascular repair and angiogenesis, while VEGF mRNA and microvessel density (MVD) were significantly higher than the uninjected group. It is worth noting that the volume and cell number of the injected stem cell suspension are easily available (1 mL of the BMSC suspension contains 107 cells/mL) [69]. Cells cultured in two dishes of 60 mm diameter can reach the culture purpose. They subsequently reported a five-year follow-up clinical trial [68]. Their result showed that the femoral head collapse rate of patients in the Ficat I-II stage was low (3 of 68 hips failed, 4.41%), but that in Ficat III was high (3 of 10 hips failed, 30%). They believed that this method was a safe and effective minimally invasive treatment strategy for patients with early ONFH [68, 70].

Li et al. investigated the natural homing ability of BMSC in the necrosis of the femoral head via peripheral vein injection. The migration directions of fluorescently labeled BMSC are observed on the ONFH model of nude mice and rabbits, but the migration mechanism still requires further studies [54]. The therapeutic effect of this method is explored in rabbit ONFH models. The femoral head cut in the longitudinal direction shows that the ONFH necrotic area remained undeveloped in the BMSC group, while the control group shows a large amount of necrotic bone. X-ray and CT results show that there was no significant change in the BMSC group, while the trabecular bone fracture and articular surface collapse occurred in the control group. Masson staining indicates that the cartilage surface of the BMSC group is not significantly exfoliated, and the necrotic area is replaced by new bone, while the control group formed a large amount of fibrous connective tissue. Osteopontin (OPN) and core binding factor 1 (Cbfa1) in the BMSC group were higher than those in the control group. They believed that bone regeneration was initiated by at least two processes after BMSC transplantation: (a) the local secretion of cytokines or growth factors to promote angiogenesis and (b) BMSC directly generating new bone [55].

CD can provide channels for in situ implantation of stem cells, and hypoxic pretreatment is a simple and feasible MSC pretreatment method. Fan et al. [71] investigated that BMSC isolated from the ONFH rabbit anterior superior iliac spine were cultured at 20% and 2% oxygen concentrations, respectively. The two groups of BMSC were evaluated after BMSC were implanted into the femoral head of rabbits via CD. The apoptotic rate, cell viability, growth factor secretion, and capillary-like structure formation of BMSC at 2% oxygen concentration were as good as those of the normal BMSC and better than those at the 20% oxygen concentration. Ciapetti et al. [72] extracted BMSC from the anterior superior iliac spine of ONFH patients and cultured BMSC at 2% and 21% oxygen concentrations. The results show that the colony forming ability of BMSC exposed to hypoxia is enhanced, with increased expressions of bone-related genes (ALP, type I collagen, and osteocalcin) and normal mineralization, compared with those cultured at 21% oxygen concentration. However, there is an opposite opinion that CD with or without stem cells will not affect the therapy efficacy of ONFH according to the analysis of many preclinical and clinical trials [73].

BMSC pretreatments with the factors above are safe, although repeated treatment is not available. However, the effects are considered to be relatively mild. Meanwhile, genetically modified cells stand out with strong and lasting interventions.

#### 3.1.3. Adipose-Derived Stem Cells and Pretreatments

The disadvantages of BMSC cannot be ignored (e.g., low stem cell yield, painful extraction procedure, and surgery complications). ADSC have attracted attention from researchers due to their high yield. ADSC induced to osteogenic differentiation can enhance osteogenesis and promote vascularization in rabbit ONFH models [74]. W9 (a peptide) can block nuclear factor-*κ*B ligand- (RANKL-) RANK signaling and enhance ADSC osteogenesis even under no osteogenic conditions [30]. VEGF 165 gene-modified ADSC also promote osteogenesis and angiogenesis [75] as well as VEGF 165-gene modified BMSC.

#### 3.1.4. Preclinical and Clinical Trials

Abudusaimi et al. [76] implanted the ADSC into the tunnel via the CD in the rabbit ONFH models. CT of the ADSC group shows an increase in trabecular bone volume and density in the necrotic area. Immunohistochemistry results showed high osteocalcin in the ADSC group. Pak [77] reported a clinical trial of 2 cases via CD with ADSC. Clinical results show improvements in their pain visual analogue scale score, physical therapy tests, and Harris Hip Score. T1-weighted MRI shows that signal changes are consistent with medullary bone regeneration.

#### 3.1.5. Other Stem Cells and Preclinical and Clinical Trials

The synovial fluid mesenchymal stem cells loaded on alginate beads have the potential of osteogenesis and differentiation. These implanted beads can help increase bone density and preserve the shape of the femoral head in rabbit ONFH models [31]. DPSC are considered to be safe cell seeds. The bone tissue restoration of DPSC therapy via CD in ovine ONFH models is better than that in heterologous MSC therapy when assessed by histological assessments [32]. Allogeneic peripheral blood-derived mesenchymal stem cells will upregulate BMP-2 expression and downregulate PPAR*γ* expression in the osteonecrosis zone. Human umbilical cord-derived MSC therapy achieved good clinical outcomes in the treatment for ONFH by femoral artery injection according to the report of Chen et al. [34]. They used the oxygen delivery index (ODI) as a prognostic indicator for ONFH and provided the calculation method (ODI = hematocrit/SBV = 100*H*/1.4175 + 5.878*H* − 12.98*H*2 + 31.964*H*3, where *H* stands for the volume fraction of erythrocytes and SBV stands for systolic blood viscosity). The indicator can be used to evaluate the oxygen transport status of the necrotic area and contribute to the ONFH assessment system to some extent. Aarvold et al. proposed the concept of SSC, although SSC was not confirmed at that time [78]. The location of SSC in the MSC differentiation pedigree was not known until Chan et al.'s [3] research on identification and characterization of SSC. Exosomes secreted by induced pluripotent stem cell-derived mesenchymal stem cells (IPSC) can reduce bone loss in the necrotic area and increase angiogenesis by activation of the PI3K/Akt signaling pathway on endothelial cells [79]. Cai et al. isolated and cultured autologous bone marrow mononuclear cells (BM-MNC) and allogeneic UCDMSC and injected mixed cell suspension by femoral artery angiography. This method presents an infusion time course of 30 minutes and a liquid volume of 90-130 mL, but no cell number. Treatment results showed that this method had a certain therapeutic effect but could not reverse the stage of ONFH patients [80].

In summary, stem cells alone with cytokines or genetic engineering techniques have not revolutionized the status of ONFH treatment. The focus is then shifted to the carriers and scaffolds.

**Figure 4:**
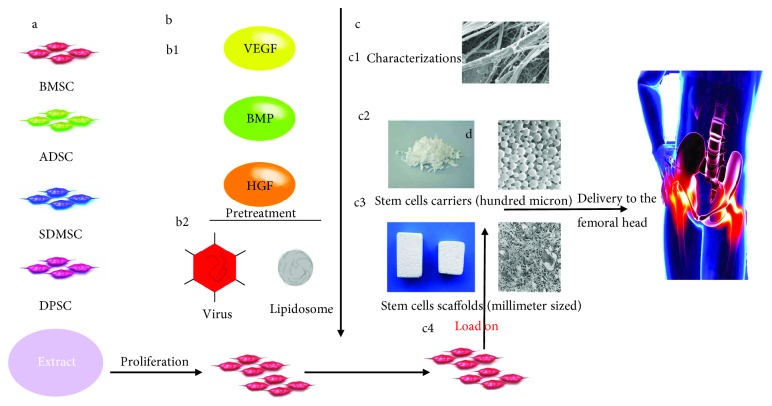
Schematic diagram of the manufacture and applications of stem cell carriers or scaffolds. (a) Stem cell population selection, extraction, and proliferation. (b) Stem cell pretreatment: (b1) the pretreatment of stem cells induced by cytokines and (b2) the pretreatment of transgenic stem cells. (c) Stem cells loaded on carriers or scaffolds: (c1) material characterization, (c2) stem cell carriers (hundred microns), (c3) stem cell scaffolds (millimeter-sized), and (c4) the pretreated stem cells loaded onto the carrier or scaffold. (d) Delivery of the stem cells loaded onto the carrier or scaffold to the femoral head necrosis area.

The carriers and support scaffolds with stem cells should be manufactured with properties of osseointegration, biodegradation, and bony replacement. Lots of efforts have been made for improvements on the three properties above. We will describe the applications of carriers and scaffolds in ONFH therapies and assess their performance in the following sections (Figure 4).

### 3.2. Stem Cells on Carriers to the Necrosis Area of ONFH

Some natural properties of carriers are preserved (e.g., the microstructure that facilitates stem cell attachment, the support strength of natural trabecular bone, and the elastic modulus prone to bone growth) following the extraction and processing, including the demineralized bone matrix (DBM) [19, 81, 82], bisphosphonate carriers [83], xenograft bone substitute [84], bone-marrow buffy coat (BBC) [85], fibrin glue [86], and small intestine submucosa (SIS) matrix. These carriers have low immunogenicity and high histocompatibility.

BMSC modified with the BMP-2 gene and basic fibroblast growth factor (bFGF) gene loaded on DBM can repair the femoral head necrotic zone in canine models, resulting in the production of a large number of new bone, the regeneration of high-density new blood vessels, and the increase in compression and bending strength [82]. Stem cells via CD have also been widely used in clinical trials. CD and MSC implants with bisphosphonate carriers probably delayed the progression of femoral head collapse in a clinical trial of 8 cases [83]. In a long-term follow-up clinical trial of 38 patients with early ONFH, 33 patients are cured in clinical outcomes and imaging manifestations through implants (autologous MSC, xenograft bone substitute, and recombinant morphogenetic proteins) via CD [84]. BBC implantation via CD can effectively prevent progress of ONFH, relieve pain, and improve joint movements for patients assessed by the Lequesne Index and Western Ontario and McMaster Universities Arthritis Index (ClinicalTrials.gov identifier NCT01613612, registered 13 December 2011) [85]. Tabatabaee et al. [87] declared that BMSC implants via CD were only effective in the early stage of ONFH. MSC mixed with medical fibrin glue were delivered to the avascular necrosis area of the femoral head, which was performed by Wen et al. [86], and they achieved good outcomes. In a HGF transgenic cell research, the results show that the therapeutic effect of the HGF-gene transgenic MSC group was superior to that of the HGF-gene transgenic fibroblast group. This phenomenon suggests that the mechanism of MSC treatment for ONFH is more inclined to MSC osteogenic theory. Song et al. [88] implanted cancellous bone, SIS, and BDMSC into the osteonecrosis area. Bone formation and angiogenesis of SIS and BDMSC are stronger than those of other combinations of interventions in the rabbit ONFH models. Better yet, this strategy has demonstrated no inflammatory cell infiltration.

In the advanced ONFH, stem cells without support scaffolds may not accomplish the treatment task. Thus, the support scaffolds appear to be particularly necessary.

### 3.3. Stem Cell Scaffolds Supporting the Femoral Head

Synthetic scaffolds are developed with spatial structure and mechanical support for ONFH therapies. Materials have been processed into scaffolds for ONFH therapies, including polylactide-co-glycolide acid (PLGA) biomimetic synthetic scaffolds [89], polylactide-co-glycolide acid and calcium phosphate (PLGA-CPC) microsphere [90] or scaffolds [91], porous tantalum rod implants [92, 93], strontium-doped calcium polyphosphate (SSCPP) [94], *β*-tricalcium phosphate (*β*-TCP) [95], and biphasic calcium phosphate (BCP) ceramic scaffolds [96].

Tantalum rods and BCP ceramic scaffolds are considered to be promising for clinical translation, owing to their good properties (e.g., high ultimate strength and Young's modulus, good osseointegration, optimal porosity, appropriate pore size, and material surface chemistry).

**Figure 5:**
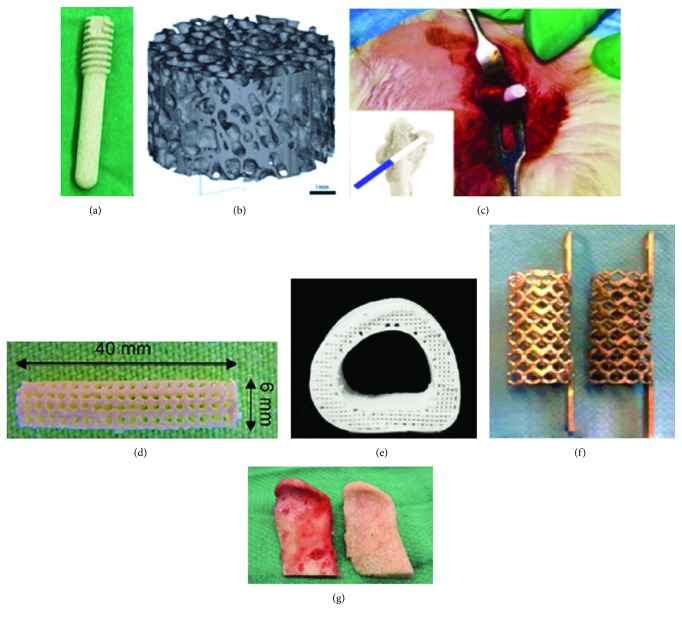
Stem cell scaffolds for ONFH treatment and other promising designs of stem cell scaffold designs. (a) The porous tantalum metal rod for supporting the necrosis of the femoral head. (b) The bone ceramic scaffold with similar dimensions as cancellous bone trabeculae (bar = 1 mm). Figure adapted from ref. [96], John Wiley & Sons Publishers Ltd. (c) The CPC scaffold loaded on microspheres (BMP and VEGF loaded on PLGA microspheres) and BMSC in the operation. Figure adapted from ref. [90], Elsevier Publishers Ltd. (d) 3D-printing PLGA/tricalcium scaffold with the polylactic acid-co-glycolic acid/tricalcium phosphate. Figure adapted from ref. [91], Elsevier Publishers Ltd. (e) Hyperelastic bone fabricated via 3D printing and liquid inks consisted of hydroxyapatite and polycaprolactone or PLGA. Figure adapted from ref. [100], The American Association for the Advancement of Science Publishers Ltd. (f) Low-elastic modulus titanium alloy 3D-printed mesh scaffold (more conducive to bone growth). Figure adapted from ref. [101], The American Association for the Advancement of Science Publishers Ltd. (g) Tissue engineered anatomical autologous bone scaffold used for facial reconstruction in high fidelity. Figure adapted from ref. [102], The American Association for the Advancement of Science Publishers Ltd.

Mao et al. [97] injected peripheral blood stem cells (PBSC) into patients' medial circumflex femoral arteries and implanted porous tantalum (Figure 5(a)) into patients' femoral head in their clinical trial (36 months of follow-up). This method provides biomechanical support and is a safe and feasible choice for patients with early- and intermediate-stage ONFH, although 3 cases required THA (48 cases in total). Meanwhile, Zhao et al. implanted porous tantalum rods with BMSC into patients' femoral head necrotic zone for the end stage of ONFH, and 5 cases failed (31 cases in total) [92, 97].

The BCP ceramic scaffolds are manufactured based on the reconstructed bone trabecula data model of microcomputed tomography images (Figure 5(b)). Peng et al. implanted the scaffolds into the necrotic area of the femoral head in dog ONFH models. The results show that the osseointegration and new bone formation of scaffolds loaded with BMSC are significantly higher than those without BMSC. Higher-strength and compressive moduli have been tested out at the repair site in the BCP and BMSC groups [96].

Kang et al. [94] doped strontium into the calcium polyphosphate (CPP) scaffolds, as strontium can inhibit bone resorption [98, 99]. Autologous BM-MNC loading on this scaffold is implanted into the necrotic zone via CD in rabbit ONFH models. The levels of VEGF expression and osteogenesis are higher than those in the CPP group and bone grafting group. Better yet, its mechanical strength is not impaired following the implantation of the femoral head.

Tomoki Aoyama and his fellows [95] implanted autologous MSC mixed with *β*-TCP granules (OSferion; Olympus Terumo Biomaterials Co., Tokyo, Japan) into patients' femoral head necrotic zone via CD in a clinical trial (UMIN Clinical Trials Registry, UMIN000001601). Some young patients with extensive necrotic lesions with pain achieved symptom relief.

Zhang et al. [90] developed a novel calcium phosphate composite (CPC) scaffold, which was loaded on microspheres (BMP and VEGF loaded on poly-lactic-co-glycolic acid microspheres) and BMSC. The scaffold has excellent characteristics, including the porosity (62%), interconnected porous structures, pore sizes (219 *μ*m), and compressive strength (6.60 MPa). These characteristics are similar to the physiological trabecular bone and indicate the good biocompatibility and osteogenesis and angiogenesis of the scaffold (Figure 5(c)).

Qin et al. [91] developed a 3D-printed scaffold with polylactic acid-co-glycolic acid/tricalcium phosphate. This scaffold is loaded with icariin and implanted into the necrotic zone via CD in both quadruped and biped animal models (Figure 5(d)). The radiographic results, gait assessments, and finite element analyses show that this scaffold can reduce the incidence of collapse, promote new bone formation, and improve hip function recovery.

Although the current scaffolds exhibit a certain therapeutic effect, their structures and properties have not been deeply optimized to the level of competence. The achievement of exact therapy efficacy for ONFH remains a challenge. The development of superior MSC scaffolds is a future goal in this field.

## 4. Conclusions and Challenges

This review outlined the emerging developments, strengths, and weaknesses of multiscale stem cell technology for ONFH from the perspective of clinicians and material developers, all of which offer significant opportunities to advance this field. We show that multiscale stem cell technology has great potential to delay ONFH progress and for bone regeneration.

Stem cell technology is rapidly evolving, and a large number of transgenic MSC for achieving specific function have been studied. The five microRNAs mentioned in Section 2 may be promising targets for improving stem cell activities. More attention should be paid to the genetic safety of stem cell subpopulations and transgenes. Strong expression of a specific factor may overly affect other normal tissues. The effects of small molecule drugs on the proliferation and differentiation of stem cells had been confirmed. However, the therapeutic cells used for ONFH are rarely investigated in this aspect. Small molecule drugs can be metabolized. Small molecule drugs appear more controllable for stem cell pretreatment and may be an alternative research direction for stem cell pretreatment for ONFH therapies in the future.

The field is rapidly evolving and has been steadily published in a variety of *in vitro* or *in vivo* animal studies or even clinical trials. However, successful goals of ONFH permanent cure via multiscale stem cell technologies are still difficult to achieve. ONFH is likely to be caused by multiple factors (e.g., ischemia, cell death, and bone resorption). The designs of carriers and scaffolds required considerations for these factors. We may draw on some design ideas from other promising methods of bone and joint regeneration materials. Besides the choice of the material types of scaffolds, the proportion of each component is a top priority in optimization. Optimizing the proportion of each component can balance the support strength and elastic modulus of the material. For example, a good bone replacement scaffold was produced by 3D printing with a surface structure suitable for stem cell growth and a good modulus for osseointegration [100] (Figure 5(e), hyperplastic “bone” designed and manufactured by Jakus et al.). In addition to the proportion of each component, we should pay more attention to the influence of the design structures of the scaffolds, thus reducing the heterogeneity on bone and joint regeneration [101] (Figure 5(f); Pobloth et al. believed that a soft titanium alloy stent grid bracket is more prone to bone growth).

The recent manufacture of tissue engineered autografts with low absorbance and high fidelity has shown very promising results in creating a new functional ball-mortar joint [102] (Figure 5(g); anatomical grafts for facial reconstruction developed by Bhumiratana et al.) Therefore, tissue engineering methods will catch our eyes in the future. The advantages of diverse technologies (3D printing technology, high-precision stereo computer numerical control engraving and stem cell technology, etc.) may be extracted and combined into a multiscale stem cell technology for tissue engineering of femoral head grafts or even the entire femoral head. Effective blood flow recanalization and bone replacement are also keys to ONFH treatment, so the establishment of the blood vessels' access to the necrotic area may result in better clinical outcomes. These design philosophies will be adopted by multiscale stem cell technologies for ONFH.

Last but not least, in order to accelerate breakthroughs in this area, funding for stem cell technology for ONFH treatment should be substantially increased. Compared to other hip treatment techniques (e.g., metal or ceramic prosthesis techniques), the femoral head necrosis stem cell technology lagged behind in breadth and depth. Its slow progress also reflects (at least in part) insufficiency in government/fund and investor investment. However, in the past few years, more funding opportunities have been created in the field of stem cell technology for ONFH treatment which has also become part of our review. We believe that multiscale stem cell technologies will revolutionize the current ONFH therapies in the foreseeable future.
